# Notes on the Intestinal Protozoal Infections of 1680 Men Examined at the University War Hospital, Southampton

**Published:** 1919

**Authors:** 


***Notes on the Intestinal Protozoal Infections of 1680 Men Examined at the University War Hospital, Southampton.*** By Doris L. Mackinson. *The Lancet,* Sept. 21, 1918.

During a period of 13 months, the stools of 1680 patients were examined for intestinal protozoa.

(1) The cases were examined as often as six times each.

(2) While 914 of them were admitted as convalescent from dysentery and severe diarrhoea, the other 766 were non-dysenterics, diagnosed typhoid, paratyphoid, trench fever, P. U. O., and so on.

(3) With less than 75 exceptions, this large body of men had been invalided from France, or had not been out of Britain.

*Number of Examinations.* Each case was examined as often as 6 times during a week before pronouncing him unaffected by E. histolytica. These numerous examinations do not prove useless, as in some cases cysts of E. histolytica are known to have been found as late as the 8th or 9th examination.

*Total Infections Detected.* Of the 1680 cases, 864 (51.4 per cent.) were found to be infected with some intestinal protozoa.

| | Number of Cases Infected. | Percentage. |
|---|---|---|
| E. histolytica | 209 | 12.4 |
| E. coli | 440 | 26.2 |
| E. nana | 303 | 18.0 |
| Giardia intestinalis | 226 | 13.4 |
| Chilomastix mesnili | 85 | 5.0 |
| Trichomonas hominis | 13 | 0.7 |

*Comparison of Infections in Dysenterics and Non-dysenterics.*

| | |
|---|---|
| Number of dysenteric cases examined | 914 |
| Number of non-dysenteric cases examined | 766 |

Both classes of patients harbored the same intestinal protozoa, the percentages being slightly higher in the dysenteric group. It seems that in non-dysenterics, with a dysenteric history, protozoal infections are rather more numerous than in cases having no such history.

*Comparison of Infections in " French " and " Eastern " Cases.*

Cases who had been in the East (i. e. tropics and those subtropical regions where amebic dysentery is usually considered to be endemic), either during the war, or at some previous time :

| | |
|---|---|
| Number examined | 447 |
| Cases never in the East | 1233 |

Comparing the incidence of the various protozoa in both groups it appears that E. histolytica as well as Trichomonas seem to be more abundant in the Eastern group.

Examination of feces of persons who had never been out of Britain has shown that all the common intestinal protozoa occur in this country.

*Size of E. Histolytica Cysts.*

Among the 209 carriers of E. histolytica with which this paper deals :

103 cases had cysts below 10.

90 cases had cysts above 10.

16 cases had mixed infections of the various sizes.

No evidences have been obtained that races of E. histolytica producing cysts of any particular size are characteristic of infections acquired in any particular locality.

*Treatment.*

E. histolytica carriers were given a course of emetine bismuth iodide in 3 gr. doses for 12 days. Such treatment had the following results :

(1) Of 131 men who had one treatment (130 for 12 days, and one for 36 days), 62 were clear for 4 weeks afterwards, and 69 relapsed.

(2) Of 16 men who had a second treatment (15 for 18 days and one for 24 days), 6 were clear for 4 weeks afterwards and 10 relapsed.

(3) Of two men who had a third treatment for 18 days, and in one case with emetine injections during 10 days of the course, 2 were clear for 4 weeks after treatment and none relapsed. At least 58 per cent. of the relapses took place in the first week.

Observations on 5 cases of " Lamblia dysentery ", treated in France with methylene blue and declared free of the organism, do not suggest that this is an effective treatment.

